# Update on Pathogenesis of Glomerular Hyperfiltration in Early Diabetic Kidney Disease

**DOI:** 10.3389/fendo.2022.872918

**Published:** 2022-05-19

**Authors:** Yang Yang, Gaosi Xu

**Affiliations:** Department of Nephrology, The Second Affiliated Hospital of Nanchang University, Nanchang, China

**Keywords:** diabetic kidney disease, glomerular hyperfiltration, sodium–glucose cotransporter, tubuloglomerular feedback, renal hemodynamics

## Abstract

In the existing stages of diabetic kidney disease (DKD), the first stage of DKD is called the preclinical stage, characterized by glomerular hyperfiltration, an abnormally elevated glomerular filtration rate. Glomerular hyperfiltration is an independent risk factor for accelerated deterioration of renal function and progression of nephropathy, which is associated with a high risk for metabolic and cardiovascular disease. It is imperative to understand hyperfiltration and identify potential treatments to delay DKD progress. This paper summarizes the current mechanisms of hyperfiltration in early DKD. We pay close attention to the effect of glucose reabsorption mediated by sodium–glucose cotransporters and renal growth on hyperfiltration in DKD patients, as well as the mechanisms of nitric oxide and adenosine actions on renal afferent arterioles *via* tubuloglomerular feedback. Furthermore, we also focus on the contribution of the atrial natriuretic peptide, cyclooxygenase, renin–angiotensin–aldosterone system, and endothelin on hyperfiltration. Proposing potential treatments based on these mechanisms may offer new therapeutic opportunities to reduce the renal burden in this population.

## Introduction

Diabetic kidney disease (DKD) is the most common cause of end-stage kidney disease, and its diagnostic standards include a decline in renal function, diabetic retinopathy, urine albumin-to-creatinine ratio increases, and a reduction in the glomerular filtration rate (GFR) (1). Due to the rising incidence of diabetes, the dominating cause of chronic kidney disease (CKD) in China has changed from chronic glomerulonephritis to diabetes-related CKD (2), with most developing from type 2 diabetes (T2DM). DKD is a heavy societal burden on society since it is detrimental to the health of affected patients. Despite lifestyle modification and the use of hypoglycemic drugs, the prevention and treatment of DKD still face severe challenges (2). One challenge to overcome is the difficulty of diagnosing DKD in its initial stages. Glomerular hyperfiltration usually occurs in an early stage of renal damage before the appearance of proteinuria (3, 4), without clinical manifestations. It is considered to be a risk factor for DKD (5). Of course, not all people with diabetes develop DKD; not everyone follows the established DKD phasing either. A Thailand study shows that nearly 40% of T2DM are already at stage 2 or worse when diagnosed with DKD (6).

Glomerular hyperfiltration is observed in type 1 diabetes mellitus (T1DM) and T2DM (7). At present, there is no clear cut-off value to define hyperfiltration (8, 9). This paper summarizes the prevalence of diabetic hyperfiltration in recent years (**Table 1**). On one hand, different methods get different results. Researchers usually used ^51^Cr-labeled ethylenediaminetetraacetic acid (^51^Cr-EDTA) to measure GFR in the 1990s (7), while its accuracy and precision are lower than technetium-99m-diethylenetriaminepentaacetic acid (^99^mTc-DTPA) clearance (22). On the other hand, biological factors, such as glycemic control, body mass index, age, sex, and ethnicity, can all influence GFR (8, 23). Notably, hyperfiltration can also occur in prediabetics whose glucose is impaired (4). In T1DM and T2DM, hyperfiltration may further exacerbate the decline in renal function, causing a faster decrease in GFR compared to non-hyperfiltration and ultimately resulting in CKD (5, 16, 19, 24). A prospective study based on 1,014 T2DM patients showed that baseline hyperfiltration is significantly associated with the odds of rapid renal decline (16). Therefore, by improving our existing knowledge about hyperfiltration, we can discover new strategies to delay the occurrence of DKD.

**Table 1:** Prevalence of diabetic hyperfiltration in recent years.

Study author(s)	Publish year	N	Total study population	GFR method	Criteria for hyperfiltration	Prevalence of hyperfiltration, %
T1DM						
Bulum et al. (10).	2013	–	313	CKD-EPI	GFR≥125 ml/min/1.73 m^2^	12
Ficociello et al. (11)	2009	104	426	Cystatin C-GFR	GFR≥134 (M)/149 (F) ml/min/1.73 m^2^	24
Molitch et al. (12)	2019	106	446	^125^I-iothalamate	GFR≥140 ml/min/1.73 m^2^	24
Naqvi et al. (13)	2016	52	121	CKD-EPI	GFR≥100 ml/min/1.73 m^2^	42.9
T2DM						
Ekinci et al. (14)	2015	29	383	Iohexol	GFR>144 ml/min/1.73 m^2a^	23.2
Jin et al. (15)	2006	16	93	Iohexol	GFR>mean GFR + 1.96 SD of control subjects^a^	17
Low et al. (16)	2018	53	1,014	CKD-EPI	GFR≥120 ml/min/1.73 m^2^	5.2
Premaratne et al. (17)	2005	110	662	^99^mTc-DTPA	GFR>130 ml/min/1.73 m^2a^	16.6
Pruijm et al. (18)	2010	–	363	Inulin	GFR>140 ml/min/1.73 m^2^	52.8
Ruggenenti et al. (19)	2012	90	600	Iohexol	GFR≥120 ml/min/1.73 m^2^	15
Shilpasree et al. (4)	2021	43	1,031	CKD-EPI	>95th percentile in normal controls^b^	8.7
T1DM+T2DM						
Hong et al. (20)	2018	809	15,918	CKD-EPI	>95th percentile in normal controls^b^	5.1
Zhao et al. (21)	2015	340	3,492	^99^mTc-DTPA	GFR>128.97 ml/min/1.73 m^2^	9.7

N, number of people with glomerular hyperfiltration; GFR, glomerular filtration rate; CKD-EPI, chronic kidney disease epidemiology; M, males; F, females; ^99^mTc-DTPA, 99mTc-diethylene-triamine-penta-acetic acid.

a After age adjusted.

b After age and sex adjusted.

To date, the most common hypothesis about the mechanisms of diabetic hyperfiltration is the tubular event (7, 25–34). This event refers to any process that affects the increase in proximal tubule glucose reabsorption and involves the upregulation of sodium–glucose cotransporters (SGLTs) and the growth of renal tubule, then through tubuloglomerular feedback (TGF) to alter GFR. Furthermore, some level of hormones and vasoactive substances are increased in people with diabetes; these substances control the contraction and dilation of the pre-glomerular and post-glomerular arteriolar, causing hyperfiltration by altering vascular resistance (7, 9, 28, 30, 35, 36). This review aims at elaborating on the interaction of the above views on hyperfiltration and discussing the potential therapies of DKD.

## Effect of Sodium–Glucose Cotransporter on Renal Glucose Reabsorption

**Figure 1:**
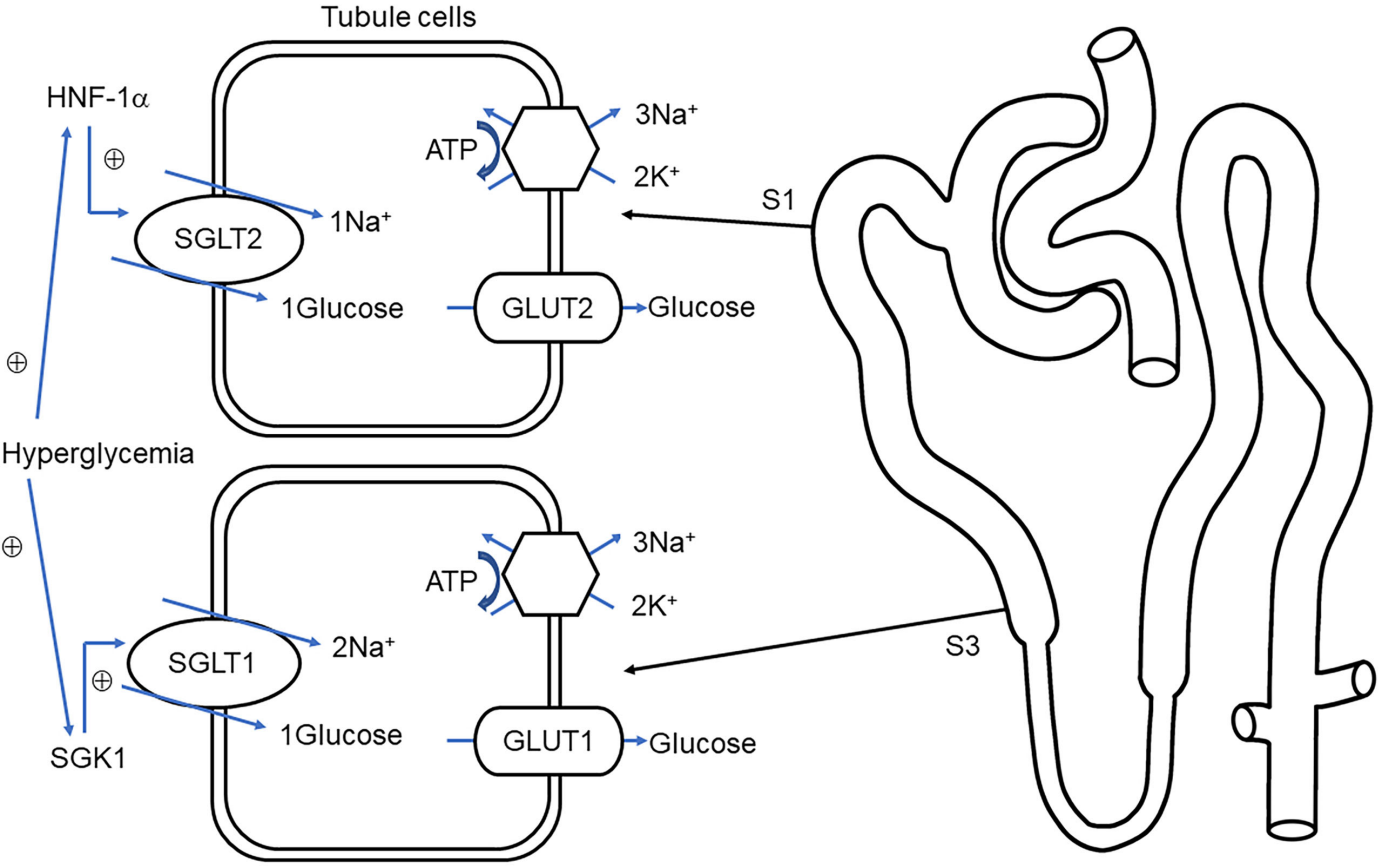
Overview of glucose transport in proximal tubules. Sodium–glucose cotransporter 1/2 (SGLT1, SGLT2) protein located on the brush border membrane is responsible for reabsorption of glucose and sodium in the early renal proximal tubule (S1) and distal proximal tubule (S3), respectively. The glucose on the S1 and S3 correspondingly combined with glucose transporter 2/1 (GLUT2, GLUT1) into the renal interstitial (38). Na^+^-K^+^-ATPase, located in the basolateral membrane maintains the potential gradient and concentration gradient of sodium ions and promotes the passive transport of sodium ions. Under hyperglycemia conditions, hepatocyte nuclear factor 1 alpha (HNF-1α), serum, and glucocorticoid-induced kinase-1 (SGK1) stimulate the upregulation of SGLT2 and SGLT1, respectively, thereby increasing glucose reabsorption (39, 40).

Nearly 180 g of glucose is filtrated by the renal glomerulus every day, and little-to-no concentration of glucose is present in the urine, which is related to the reabsorption of the renal tubule. Glucose appears in urine when filtered glucose surpasses the maximum renal absorptive capacity. SGLTs are present in proximal tubules (37) where sodium ions traveling intracellularly along the electrochemical gradient carry glucose molecules in the same direction. Next, glucose is transported to the renal interstitial fluid through glucose transoporters (GLUTs) by the way of facilitated diffusion and eventually backflow into the bloodstream (**Figure 1**).

There are two dominating SGLTs in the proximal tubule that are responsible for glucose reabsorption, SGLT2 and SGLT1 (41). Evidence indicates that the renal reabsorption of glucose was significantly lower in SGLT2 knockout mice (42) and their urinary glucose excretion is significantly higher (43). David et al. demonstrated that the 24 h urinary glucose excretion of SGLT1 and SGLT2 knockout mice accounted for ~2% and ~30% of the double gene knockout mice, respectively (44). Further evidence suggests that familial renal glycosuria is caused by the individuals’ mutations of the SGLT2 gene, which is featured with persistent glycosuria without hyperglycemia or any symptom of renal tubular dysfunction (45). On the contrary, SGLT1 mainly mediates intestinal glucose absorption, so genetic defects in SGLT1 are more likely to cause neonatal diarrhea and rarely with glycosuria (38, 46). In summary, low-affinity, high-capacity SGLT2 localized at the brush border of the early proximal tubule plays a major role in glucose reabsorption (43, 47) while high-affinity, low-capacity SGLT1 plays an indispensable role in complementing SGLT2 in distal proximal tubule-mediated glucose transport (42).

Different from SGLT1 and SGLT2, it has been shown earlier that SGLT3 is not a glucose transporter but a glucosensor (48). One study showed SGLT3 overexpression in HK-2 cells in diabetic patients, which increased intracellular Na^+^ levels and induced diabetic hyperfiltration and kidney injury (49). Both SGLT4 and SGLT5 are thought to be kidney mannose and fructose transporters. Meanwhile, SGLT5, also involved in controlling glucose reabsorption, may be related to the level of 1,5-anhydroglucitol (50). The location of SGLT6 in the kidney is not clear; it primarily transports myo-inositol rather than glucose (51). SLC5A11, encoding SGLT6, may play a role in human autoimmune diseases by interacting with immune genes (52).

## Tubular Mechanisms for Hyperfiltration in Early Diabetic Kidney Disease

### Increased Reabsorption

People with diabetes typically have higher blood glucose in comparison to normal people, and to balance homeostasis, more glucose needs to be reabsorbed to ensure the absence of glycosuria, which primarily depends on the upregulation of SGLTs and GLUTs (53–55). When comparing the diabetic rats to the normal ones, the mRNA of SGLT2 and SGLT1 was increased by 36% and 20%, respectively (56). Other evidence demonstrates that more expression of SGLT2 is present within the urine from a human who has non-insulin-dependent diabetes (57). With the increases of renal tubule reabsorption, the sodium and chloride delivered to the macula densa decreases; reducing the signal for TGF leads to the elevation of the single nephron glomerular filtration rate (SNGFR) (25, 32). The published concentration of sodium chloride on the macula densa of the diabetic nephron is less than normal (25, 29), which shows an evidence of hyperreabsorption.

Excessive glucose stimulates the transcription level of serum and glucocorticoid-induced kinase-1 (SGK-1), which, in turn, excites many carriers including the Na^+^/H^+^ exchanger NHE3, SGLT1, GLUT1 to decrease glycosuria (39, 58). In the SGK-1-knockout mice, the excretion of urinary glucose is higher (58). Moreover, SGK1 is also involved in promoting the fibrosis of diabetic kidneys (39). Similarly, enhanced SGLT2 mRNA in diabetes is positively correlated to the activation of hepatocyte nuclear factor 1 alpha (HNF-1α) (40). This observation was supported, when subsequent data showed that the expression of SGLT2 is lower in HNF-1α-null mice (59). Hence, there is reason to believe that SGK-1 and HNF-1α have a certain impact on the development of DKD. At the same time, excessive filtration increases oxygen consumption; continuous reabsorption further worsens tubular hypoxia and tubulointerstitial fibrosis (60).

The contribution of a newly oral antidiabetic drug, SGLT2 inhibitors (SGLT2is), has been well established in T1DM and T2DM. It recovers the signal of TGF by increasing the concentration of sodium in macula densa, ameliorating glomerular hypertension and hyperfiltration to the same extent as the tubular hypothesis (61). Reducing the glucose levels is the vital pharmacologic action that it provides. The reduction of hyperglycemia mitigates renal tubular transport; it also reduces renal oxygenation at the corticomedullary junction, resulting in the accumulation of hypoxia-inducible factors, which is beneficial for the production of serum erythropoietin (34). In addition, SGLT2is also lead to weight loss and a drop in blood pressure, thereby significantly reducing the incidence of cardiovascular events (34). Existing clinical trials have demonstrated that SGLT2is reduce the risk of renal failure and cardiovascular outcomes (62–65). A multinational observational cohort study showed 114 and 237 cases of eventual renal outcomes after the application of SGLT2is and other hypoglycemic agents, respectively, during the follow-up period, with the former showing a significantly lower risk (65). Notably, most trails enrolled patients whose GFR is low, with few early-stage patients (62–64). Future studies of SGLT2is should target more patients with an early stage of DKD; after all, hyperfiltration is a cause of DKD progress.

While SGLT1, as a main protein carrier of intestinal glucose absorption, also has great therapeutic potential (66), a dual SGLT1/2 inhibitor, licogliflozin, reduces blood glucose by changing urinary glucose excretion and hemodynamics decline (67). Larger and longer clinical trials are needed to investigate the long-term safety, efficacy, and potential beneficial effects of this drug.

### Kidney Growth and Salt Paradox

At the onset of diabetes, kidneys grow due to expanded nephron size; renal hypertrophy occurs mainly in the cortex of the diabetic kidney and is linked to subsequent proximal tubular hyperreabsorption (25, 55). Uehara−Watanabe et al. showed that the kidney weight, proximal tubules, and glomeruli size were significantly higher in streptozotocin-induced rats (55). Greater glomerular volume and glomerular basement membrane (GBM) width were also demonstrated in patients with early DKD (68). The elevated surface density of peripheral GBM and glomerular filtration surface area are closely related to glomerular hyperfiltration (68). Kidney growth is most likely caused by various cytokines and growth factors stimulating various signal pathways to respond to hyperglycemia, including the transforming growth factor-β (TGF-β) system, which is crucial for mesangial cell hypertrophy, fibrosis, and glomerulosclerosis (69). The vascular factor and protein glycation products amplify the expression of TGF-β on diabetes (69), from cell proliferation to hypertrophy to cellular senescence (28, 29, 31). It is well known that hyperglycemia plays a key role in the progression of DKD by activating advanced glycation end products (AGEs), protein kinase C (PKC), and the aldose reductase pathway (70). Cell growth, fibrosis, and tissue damage are related to increased PKC activity, especially in diabetes (71). In a diabetic mice model, the selective inhibitor of PKC-β, ruboxistaurin, amelioration overexpression of TGF-β improved glomerular hyperfiltration and reduced albuminuria (71, 72). Ruboxistaurin has been certified as beneficial to DKD therapy in a short time of clinical studies but not in long-term studies (72).

Based on a renal tubule growth phenotype, researchers found an inverse relationship between dietary NaCl intake and GFR, which is called salt paradox (73). In other words, the lower the NaCl intake of diabetics, the higher their GFR. The possible explanation is that after high salt intake, the concentration of sodium chloride reaching the macula densa is increased; the TGF signal is reactivated, which reduces SNGFR (26, 73). Persistent hyperglycemia makes reabsorption increase sensitivity to dietary NaCl (73). The activity of ornithine decarboxylase (ODC) is increased many times in diabetic rats (32, 74); it is required for the salt paradox (75). After using an ODC inhibitor, the proximal tubule hyperresponsiveness to salt was remedied, tubular growth was limited, and proximal reabsorption was attenuated, all of which improved hyperfiltration (32, 75). Therefore, ODC is necessary for renal growth and salt paradox.

### Adenosine–Tubuloglomerular Feedback

TGF is a negative feedback system, which is modulated by sensing the concentration of sodium chloride in the macula densa (76, 77). TGF is a major controller of GFR changes in the tubular hypothesis (27, 30, 31). The physiological function of TGF is reliant on the adenosine 5’-triphosphate (ATP) and adenosine (76, 77). When the macula densa detects an increase in sodium chloride concentration in the tubule fluid, it stimulates ATP hydrolysis to adenosine and extracellular release, which, in turn, acts on A1 adenosine receptors (A1AR) in afferent arterioles. TGF can then be activated, leading to the contraction of the afferent arteriole followed by decreased GFR (31, 76). Therefore, the role of adenosine cannot be ignored, but the exact effect of A1AR for TGF on diabetic hyperfiltration has yet to be clarified. In some A1AR knockout diabetic mice, the degree of diabetic hyperfiltration was the same as the control groups (78, 79), which does not support TGF-mediated increase in GFR. Another point of view is that A1AR knockout diabetic mice reduce the activity of TGF (80, 81) and ameliorates hyperfiltration (82). The difference between two viewpoints is the interference of confounding factors like blood pressure and severe hyperglycemia (29). The use of empagliflozin validates the response of A1AR to TGF (83).

Except for A1AR-mediated vasoconstriction in the low concentration range, the adenosine diphasic response has been shown in the isolation and perfusion of renal glomerular arterioles (84). Another subtype of adenosine receptor A2, involving A2aAR and A2bAR, mediates vasodilation at higher concentrations (76). Under normal circumstances, compared to A2bAR, A2aAR has a higher affinity for efferent arterioles and preferentially dilates the efferent arterioles, maintaining GFR in a normal range (85, 86). Interestingly, in diabetes, the effect of adenosine A2aAR is diminished, increasing the resistance of efferent arterioles leading to the elevation of the filtration fraction and GFR (86, 87). Recently, Patinha et al. demonstrated that the decrease in plasma glucose, reduction in proteinuria, and improvement in renal fibrosis in diabetic mice may be associated with the upregulation of A2aAR, which may serve as a promising therapeutic target for hypertension-DKD (88). Furthermore, the absence of A1AR does not influence the effect of TGF changes on the activation of the A2aAR on the efferent arterioles (31). Whether hyperfiltration can be controlled by stimulating A2aAR in the clinic, it may be a new alternative treatment that can delay the progression of DKD; more experiments must be conducted to confirm this.

In addition, A2bAR has a pathogenic effect in early glomerular dysfunction in diabetic rats (89). An experiment model shows the A2bAR-mediated overproduction of the vascular endothelial growth factor (VEGF) in early DKD rats (89), which is associated with hyperfiltration, proteinuria, and the ultrastructural changes of glomerulus (90). VEGF also promotes endothelial cell damage, which is the first barrier to the glomerular filtration membrane and may lead to the production of microalbuminuria (91). However, VEGF is an important mediator in the recovery from other kidney diseases (92). A2bAR blockers may be a novel alternative for the treatment of DKD patients.

### SGLT1-NO Synthesis1 Pathway

NO was thought to be a powerful vasodilator (93). The increased expression of NO, which is associated with hyperfiltration, was reported in diabetic kidneys (94–96). Zhang et al. used the selective GLUT1 antagonist and NOS inhibitor, respectively, to block the response of the vascular and afferent arterioles to hyperglycemia successively; it indicated that vasodilation induced by hyperglycemia is achieved through the GLUT1-mediated endothelium-dependent production of NO (97).

Recent research has proposed a novel idea that increased production of NOS-dependent NO in the macula densa results from sensing elevated glucose concentration in the luminal of macula densa through SGLT1, thereby reducing TGF activity resulting in hyperfiltration (98–100). Raising GFR through the SGLT1-NOS1-GFR pathway maintains the urinary sodium and fluid excretion and volume balance (31). Normally, the concentration of glucose in luminal can be ignored; however, in a high-sugar environment or the use of the inhibitor of SGLT2, there is an elevated glucose concentration at the lumen of the macula densa when filtrated glucose exceeds the maximum reabsorption capacity. One study found that elevated GFR induced by acute hyperglycemia was significantly attenuated in mice without NOS1 (99). There was another experiment with SGLT1 knockout mice, whose GFR, kidney weight, glomerular size, and proteinuria were reduced (98). Those discoveries established the decisive factor of macula densa NOS1 and SGLT1 in glomerular hyperfiltration related to high glucose and may provide evidence for potential new therapeutic targets, but whether it exists in chronic mild hyperglycemia is not clear; after all, increased glucose delivery to macula densa is generally found only in the medium-to-high blood glucose situations.

**Figure 2:**
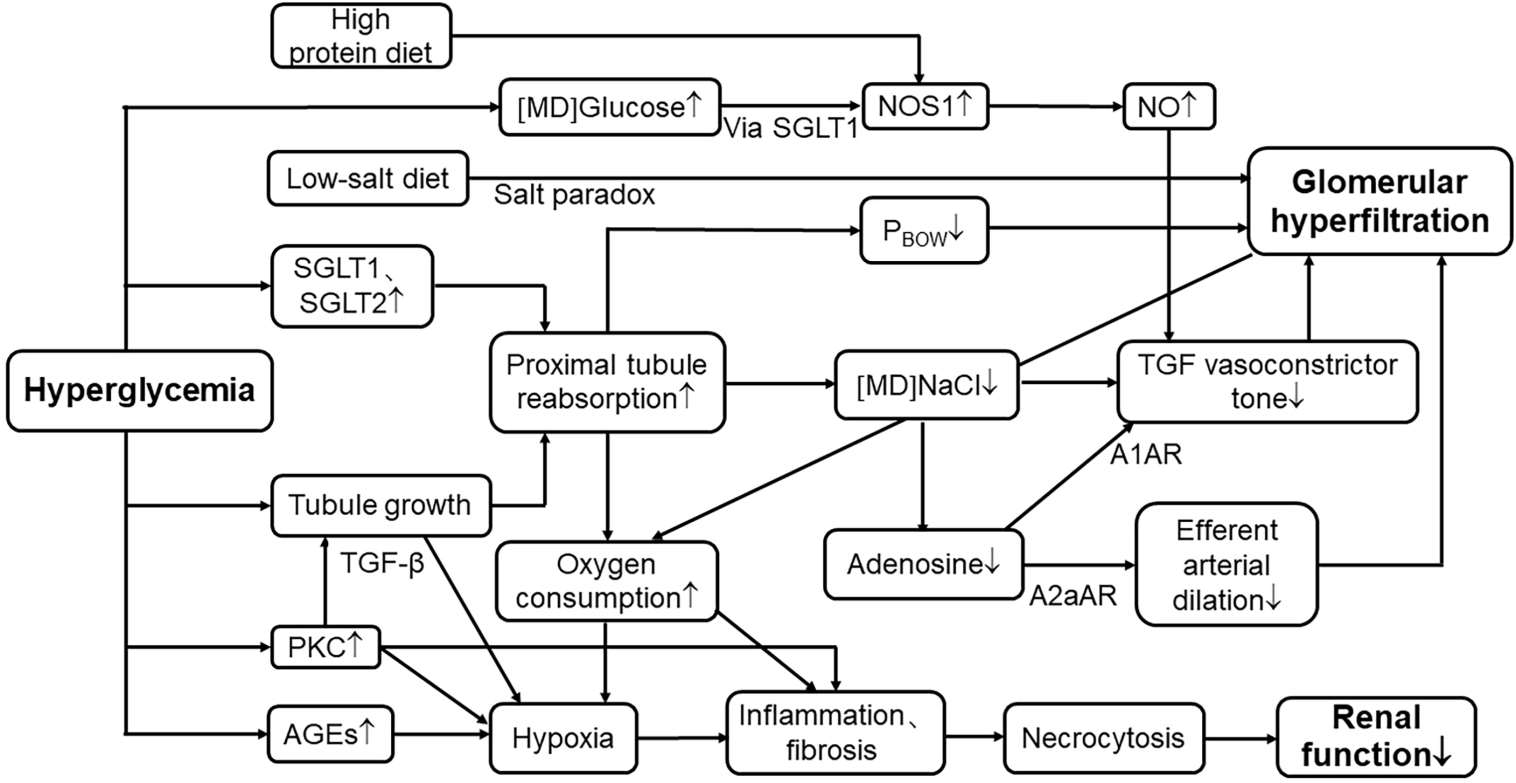
Mechanisms of hyperglycemia in renal tubular events leading to hyperfiltration and progression of nephropathy. SGLT1, sodium–glucose cotransporter1; SGLT2, sodium–glucose cotransporter2; [MD]NaCl, the concentration of sodium chloride in macula densa; TGF, tubuloglomerular feedback; P_BOW_, hydraulic pressure in Bowman’s space; [MD]Glucose, the concentration of glucose in macula densa; NOS1, nitric oxide synthase1; NO, nitric oxide; A1AR, A1 adenosine receptor; A2aAR, A2 adenosine receptor; PKC, protein kinase C; AGE, advanced glycation end products; TGF-β, transforming growth factor-β; ↑, elevate or increase; ↓, decrease or reduce.

Meanwhile, this pathway can also be activated after a high-protein meal (101). According to a retrospective cohort study of forty-three kidney donors in Tokyo, Oba et al. found that high protein intake is positively related to SNGFR and leads to hyperfiltration (102). They measured GFR at the level of a single nephron as a representative parameter on renal hemodynamic change (102), avoiding the number of functioning glomeruli affecting total renal filtration. Compared to animal protein, plant-sourced protein shows a strong beneficial effect in DKD (103). Thus, diabetic patients should not only strictly limit their sugar intake but also animal protein intake to avoid accelerating the progression of this disease (**Figure 2**).

## Vascular Mechanisms for Hyperfiltration in Early Diabetic Kidney Disease

### Cyclooxygenase

The COX-metabolite of arachidonic acid is prostaglandins (PGs); it plays a pivotal role in regulating the renal blood flow and GFR. Several observations in the diabetic mice model have verified that renal PGs are increased, including prostaglandin E_2_ (PGE_2_), prostaglandin I_2_ (PGI_2_), prostaglandin F2 alpha (PGF_2α_), and thromboxane B_2_ (104, 105). Increased PGs alters diabetic renal hemodynamics in a way of dilating renal afferent arterioles. Increased glomerular capillary pressure heightens the tensile stress on the capillary wall, resulting in an increase of the length and area of GBM, while an increased ultrafiltrate, in turn, elevates the shear force on the podocyte foot processes and body surface (106). Both mechanical stresses eventually lead to podocyte shedding, extensive GBM exfoliation, and segmental sclerosis, exacerbating kidney function damage (106).

Indomethacin, a nonselective COX inhibitor, partially reduced GFR (107). Further experimental demonstrated that COX2 inhibitors significantly reduce GFR; despite the lack of normalization (108), but selective COX1 inhibitors did not affect renal hemodynamics in diabetic rats (109). Nevertheless, Craven et al. considered that the hyperfiltration mediated by PGs occurred within 2 weeks of streptozotocin-induced diabetic mice; PG production did not increase after 4 weeks, so persistent hyperfiltration was not mediated by PGs, suggesting that sustained effects may be mediated by other factors (105). COX2 is a pivotal component in stimulating renin release by the macula densa, possibly through the release of renin-stimulating PGE_2_ and PGI_2_ (110). Consistent with this view, the level of renin decreased in COX2 knockout mice, suggesting that the PGs produced by COX-2 could affect the renal hemodynamic balance by regulating the activity of the renin–angiotensin–aldosterone system (RAAS) (111).

### Atrial Natriuretic Peptide

ANP can cause the dilation of renal afferent arteriolar and constriction of efferent arteriolar (112) and was hypothesized to be a potential mediator of diabetic hyperfiltration as early as the 1980s (113). In diabetic mice, afferent arteriolar resistance is reduced more than the efferent to increase intraglomerular pressure and GFR (107). ANP contributes to the hyperfiltration as documented by the elevated level of plasma concentration in diabetic mice and patients and by the reduction of GFR that followed the injection of the ANP-specific antiserum or antagonist (107, 114, 115). Liu et al. showed that the level of plasma ANP is significantly associated with the secretion of cytokines, which promotes the progression of DKD (115). Moreover, ANP also increases urinary albumin excretion in normoalbuminuric T2DM (116). The dual effect of ANP on hyperfiltration and proteinuria may be a predictor of the development of DKD in the long term.

However, on the other hand, ANP showed benefit in preventing and reversing kidney injury. Sacubitril/valsartan, as a dual inhibitor of neprilysin and the angiotensin II (Ang II) receptor, strengthens the effect of ANP and mitigates the hyperfiltration and renal tubular injury in the animal models of early DKD (117). This may counteract the dilated effect of ANP due to the inhibition of the vasoconstrictive effect of Ang II, resulting in a slight decrease in intraglomerular pressure and thus a protective effect on the kidney. In addition, the effect of this drug is limited to patients with heart failure (118). Therefore, more studies are needed in diabetic patients without heart failure or varying stages of DKD to explore the prognosis of this drug on the kidney.

### Renin–Angiotensin–Aldosterone System

Regarding the intrarenal hemodynamic alterations, the role of RAAS cannot be ignored (119–121). Ang II, which is the key substance in RAAS, mediates the contraction of the afferent and efferent arteries. When RAAS is activated, it causes intraglomerular pressure rises, damages tissue, stimulates fibrosis, promotes the mesangial matrix increases, and ultimately leads to diabetic glomerulosclerosis (121, 122). The SGLT2is also decreased the activation of RAAS to reduce hyperfiltration (61). Notably, after the use of the RAAS inhibitor, GFR did not fall to normal levels. This was due to the vasoconstriction effect of Ang II that may be regulated by a vasodilator, including NO and PGs, which also have an influence on hyperfiltration (123). One of the effects of sacubitril/valsartan described above is the inhibition of the Ang II receptor, which influences hemodynamic changes by dilating the efferent arterioles. It has become a hot topic of research due to its reduced risk of renal insufficiency compared with RAS inhibitors alone (124).

Furthermore, angiotensin 1-7 (Ang1-7)-mediated production by angiotensin- converting enzyme2 is also associated with hyperfiltration (125). Ren et al. found that Ang1-7 has vasodilation on afferent arteriole in isolated rabbits, further increases the intraglomerular pressure as well as stimulus production of PGs and NO (126). This function eliminated by the NOS inhibitor rather than COX means that the vasodilatory effect of Ang1-7 is dependent on the production of endothelial NO (126). Until now, RAAS inhibitors have been the gold standard therapy in DKD, since on one hand, they reduce efferent arterial contraction and blood pressure and thereby improve hyperfiltration (127). On the other hand, they prevent fibroblast activation, which delays the development of nephropathy (128).

### Endothelin

**Figure 3:**
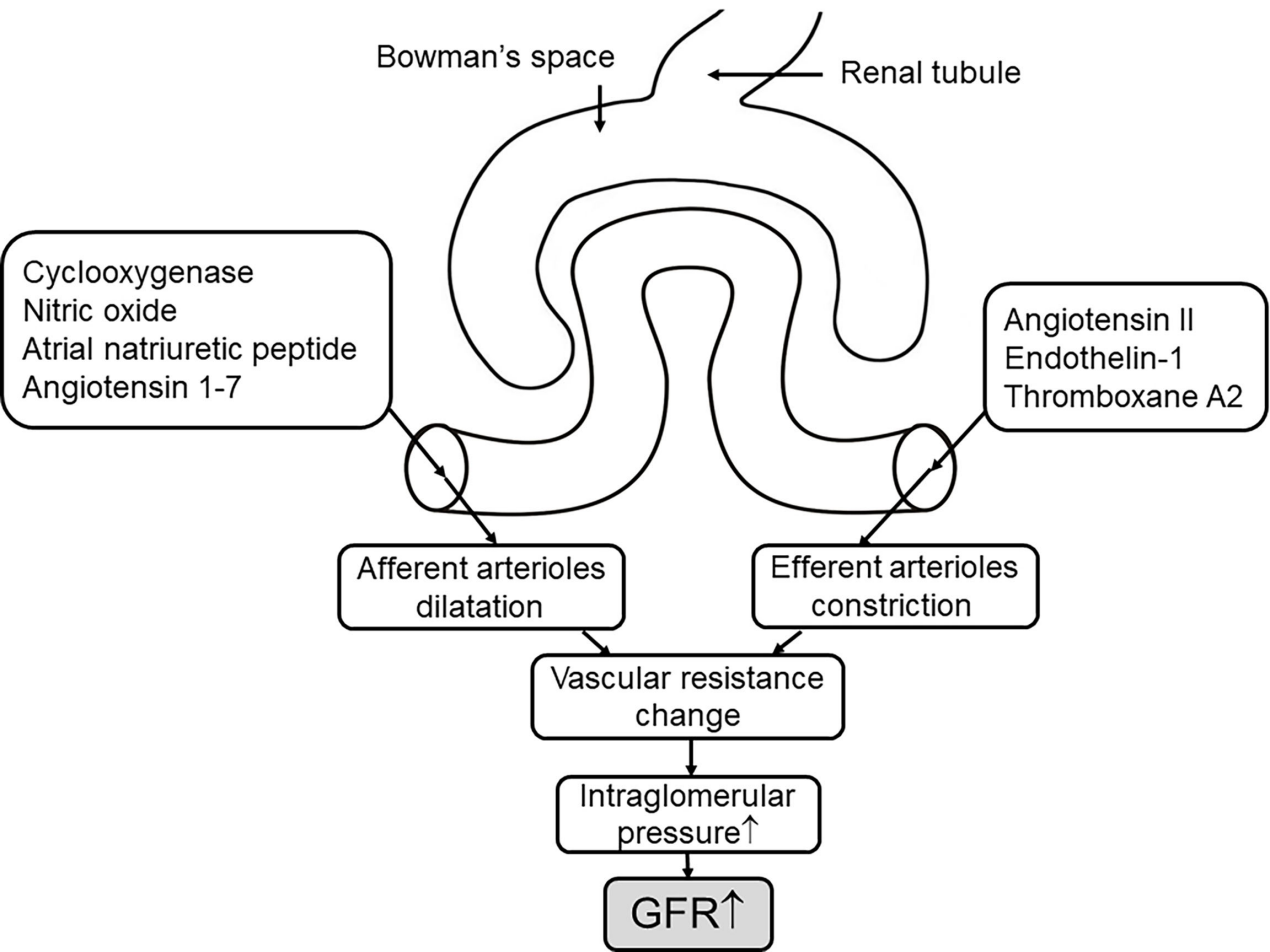
The mechanisms of the vascular events on hyperfiltration. The concentration of hormones and vasoactive mediators that mediate afferent arteriole dilation and efferent arteriole constriction increase in response to hyperglycemia (7) through altering the vascular resistance to elevate the pressure of intraglomerular, thus leading to increased GFR. ↑, elevate or increase.

Another endodermal material, endothelin-1 (ET-1), is vital in maintaining the homeostasis of sodium and water, as well as controlling the glomerular vascular tone and hemodynamics (129). Like Ang II, ET-1 can induce inflammation and prompt fibrosis (129); both factors are involved in diabetic kidney growth that is associated with the activation of TGF-β (69). ET-1 combined with ET_A_ receptor located on the vascular smooth muscle stimulates vascular constriction, whereas the release of NO and PG triggers vasodilation through the ET_B_ receptor (129, 130). Under normal conditions, the small amount of ET produced by vascular endothelium has little effect on systemic hemodynamics (131). Elevated plasma ET-1 levels have been found in T2DM, which leads to endothelial dysfunction (132). Recently, in a three-clinical-trial analysis, the kidney hemodynamic profile in adults with type 2 diabetes showed that endothelial dysfunction is associated with glomerular hyperfiltration (133). Evidence suggests that the vasoconstrictive effect of endothelin is amplified in DKD (129, 130). Renal vascular resistance and filtration fraction increased compared with the control group after exogenous ET administration, which indicates the presence of renal hyperfiltration (35, 131). Endothelin-receptor antagonists have been shown in several clinical trials and experimental models to prevent diabetic hyperfiltration, reduce proteinuria, and delay the progression of renal damage (134–136). Despite some complications of this antagonist, more trials are being explored with the promise of this new drug treating DKD (121) and whether it has greater pharmacological benefits in combination with drugs such as SGLT2is (**Figure 3**).

## Conclusion

With increasing attention to the DKD, there is growing evidence that hyperfiltration affects the progression of DKD. It occurs through a variety of mechanisms, including glucose reabsorption mediated by SGLTs, renal growth, the adenosine signal, and SGLT1-NOS1-pathway; all of these elevate GFR through weakened TGF signaling. While vasoactive mediators cause changes in GFR by altering the vascular resistance of the afferent and efferent arterioles, we attempt to propose underlying therapies to improve hyperfiltration. Clinical trials have demonstrated the renal protective effect of SGLT2is and RAAS inhibition. Upregulated A2aAR, PKC-β inhibitors, and endothelin-receptor antagonists provide new ideas for delaying the progression of DKD, but their limitations also need to be considered. Further studies of renal prognosis are needed to assess the long-term effectiveness and safety of these strategies.

## Author Contributions

YY: conceptualization, software, writing—original draft. GX: idea, funds, and paper revision. All authors contributed to the article and approved the submitted version.

## Funding

This work was supported by the National Natural Science Foundation of China (No. 81970583 & 82060138), the Nature Science Foundation of Jiangxi Province (No. 20202BABL206025), and the Projects in the Second Affiliated Hospital of Nanchang University (No. 2019YNLZ12008).

## Conflict of Interest

The authors declare that the research was conducted in the absence of any commercial or financial relationships that could be construed as a potential conflict of interest.

## Publisher’s Note

All claims expressed in this article are solely those of the authors and do not necessarily represent those of their affiliated organizations, or those of the publisher, the editors and the reviewers. Any product that may be evaluated in this article, or claim that may be made by its manufacturer, is not guaranteed or endorsed by the publisher.

## Abbreviations

A1AR, A1 adenosine receptors; A2aAR, A2bAR, A2 adenosine receptors; AGE, advanced glycation end product; Ang II, angiotensin II; Ang1-7, angiotensin 1-7; ANP, atrial natriuretic peptide; ATP, adenosine 5’-triphosphate; CKD, chronic kidney disease; COX, cyclooxygenase; ^51^Cr-EDTA, ^51^Cr-labeled ethylenediaminetetraacetic acid; DKD, diabetic kidney disease; ET-1, endothelin-1; GBM, glomerular basement membrane; GFR, glomerular filtration rate; GLUT, glucose transporter; GLUT1, glucose transporter 1; HNF-1α, hepatocyte nuclear factor 1 alpha; ^99^mTc-DTPA, technetium-99m-diethylenetriaminepentaacetic acid; NO, nitric oxide; NOS1, NO synthesis 1; Nrf2, nuclear factor erythroid 2-related factor 2; ODC, ornithine decarboxylase; PGE_2_, prostaglandin E_2_; PGF_2α_, prostaglandin F2 alpha; PGI_2_. prostaglandin I_2;_ PG, prostaglandin; PKC, protein kinase C; RAAS, renin–angiotensin–aldosterone system; SGK-1, serum and glucocorticoid-induced kinase-1; SGLT1, sodium–glucose cotransporter 1; SGLT2, sodium–glucose cotransporter 2; SGLT2i, SGLT2 inhibitor; SGLT3, sodium–glucose cotransporter 3; SGLT4, sodium–glucose cotransporter 4; SGLT5, sodium–glucose cotransporter 5; SGLT6, sodium–glucose cotransporter 6; SGLT, sodium–glucose cotransporter; SNGFR, single nephron glomerular filtration rate; T1DM, type 1 diabetes mellitus; T2DM, type 2 diabetes mellitus; TGF, tubuloglomerular feedback; TGF-β, transforming growth factor-β; VEGF, vascular endothelial growth factor.
